# Highlights from the ISCB Student Council Symposium 2013

**DOI:** 10.1186/1471-2105-15-S3-A1

**Published:** 2014-02-11

**Authors:** Tomás Di Domenico, Cynthia Prudence, Esmeralda Vicedo, Emre Guney, Anupama Jigisha, Avinash Shanmugam

**Affiliations:** 1 Department of Biology, University of Padua, Padua, Italy; 2 Département de Biologie Moléculaire Végétale, Université de Lausanne, Lausanne, Switzerland; 3 Department for Bioinformatics and Computational Biology, Institut für Informatik, TU München, Munich, Germany; 4 Center for Complex Network Research and Department of Physics, Northeastern University, Boston, MA, USA; 5 Centre Hospitalier Universitaire Vaudois, Université de Lausanne, Lausanne, Switzerland; 6 Department of Computational Medicine and Bioinformatics, University of Michigan, Ann Arbor, MI, USA

## Abstract

This report summarizes the scientific content and activities of the annual symposium organized by the Student Council of the International Society for Computational Biology (ISCB), held in conjunction with the Intelligent Systems for Molecular Biology (ISMB) / European Conference on Computational Biology (ECCB) conference in Berlin, Germany, on July 19, 2013.

## About the Student Council and the symposium

The Student Council (SC), part of the International Society for Computational Biology (ISCB), aims at nurturing and assisting the next generation of computational biologists. Our membership and leadership are composed of volunteer students and post-docs in computational biology and related fields. The main goal of our organisation is to offer networking and soft skill development opportunities to our members.

The Student Council Symposium (SCS) takes place every year, directly preceding the ISMB/ECCB conferences. SCS 2013 marked the ninth consecutive edition of the event, after the success of previous years' meetings in Long Beach 2012 [1], Vienna 2011 [2], Boston 2010 [3], Stockholm 2009 [4], Toronto 2008 [5], Vienna 2007 [6], Fortaleza 2006 and Madrid 2005.

## Meeting format

The Student Council Symposium is a one-day event. As is now a tradition, SCS 2013 began with a scientific speed dating session. During this session our delegates have to find a partner to introduce themselves to, and they must learn a bit about each other's scientific background. They must switch partners and repeat the process every two minutes until the allotted time runs out. The traditional scientific component of the meeting consisted of three oral presentation sessions, each with a keynote talk and several student presentations. During coffee breaks and the dedicated evening poster session participants had the opportunity to network and discuss their work.

At SCS2013, Dr. Alex Bateman (European Bioinformatics Institute, UK), Prof. Satoru Miyano (University of Tokyo, Japan) and Dr. Gonçalo Abecasis (University of Michigan, US) generously agreed to deliver the keynote addresses. In addition, Dr. Cheng Soon Ong gave a short presentation about the research activities at our institutional partner NICTA (Australia).

SCS 2013 received 97 submissions from students, which were peer-reviewed by 24 independent reviewers. Approximately 50 abstracts were accepted for poster presentations, and 10 abstracts were invited to deliver an oral presentation. Abstracts of oral presentations are included in this report. All abstracts are available online in the SCS 2013 booklet (http://iscbsc.org/scs2013/content/booklet.html).

## Keynotes

Dr. Alex Bateman's keynote opened the day, introducing us to the world of molecular biology databases in the 21^st^ century. Dr. Bateman made a case for the importance of the biocurators' role in modern biological research, considering them to be the “unsung heroes of biology who order and make sense of the immense primary literature for us”. In the second part of his talk he gave our delegates an overview of his career path, which included some invaluable advise for young researchers hoping to make their way into a successful research career.

Prof. Satoru Miyano kicked off his presentation after the lunch break by establishing an amusing parallelism between Japanese politics and the development of cancer. He then went on to describe his efforts on shedding light on cancer gene networks by using the impressive capabilities of the Human Genome Center's supercomputer, located at the University of Tokyo.

For the last keynote presentations we were proud to present the 2013 Overtone Prize winner, Dr. Gonçalo Abecasis. Dr. Abecasis gave an insightful overview of the advances achieved during the last 10 years of developments in human genetics, and the role that computational biologists played in such endeavours. He then reviewed the challenges and opportunities that the future of the field holds, putting emphasis on the contributions that young computational biologists are in the position to make.

## Workshop

Dr. Thomas Abeel, from the Broad Institute of MIT and Harvard, presented a workshop entitled “Presenting your science visually”. During the workshop, Dr. Abeel gave some insight on the available techniques to make data presentations more effective. With illustrative examples, which applied concepts ranging from the Gestalt principles and colour theory to simple tips on how to stand in front of an audience, the presenter brought awareness to the young researchers in the audience about the importance of attending to detail when communicating your results.

## Student presentations

In the first student presentation, Shanmugasundram et al. [7] introduced the LAMP database of apicomplexan metabolic pathways (http://www.llamp.net). The resource makes available to the community the annotated metabolic pathways of eight apicomplexan species, together with a comparative analysis of their metabolisms.

Saccharomyces cerevisiae's gene regulation undergoes major changes in order for the organism to adapt to environmental stress. In their work, Chasman et al. made use of integer linear programming to try to understand the complex signalling network underlying these changes.

Aiming towards a better understanding of the Nonsense mediated mRNA decay (NMD) surveillance pathway, Kahles et al. created a pipeline which constitutes a transcriptome wide, splicing sensitive analysis of NMD in plants. Their results suggest that NMD plays a major role in shaping the transcriptome.

Currently, many computational methods for the conformational analysis of proteins are mesh-based. This makes them incur in the so-called “curse of dimensionality”, since their computational cost increases exponentially with the number of atoms in the analysed molecule. Lie [8] proposed a mesh-free method which proves to be on-par with its mesh-based alternatives, while greatly lowering the computational cost involved.

Affeldt et al. [9] analysed the expansion of “dangerous” gene families implicated in a variety of genetic diseases. They argue that this expansion is a consequence of the families' susceptibility to deleterious mutations and of the purifying selection of post-whole-genome duplication species, and perform a robust statistical analysis to back their claims.

Striving towards a better understanding of the molecular mechanism of the metastatic process in breast cancer, Engin et al. used protein structure and networks at the system level to unravel the complexity of the disease's genotype-phenotype relationship. Their findings may help improve clinical methods for approaching this disease, which the American Cancer Society ranks as the second cause of cancer death among women.

Far from being static entities, proteins often exist in an equilibrium of conformations. Narunsky et al. [10] presented ConTemplate, an automated method to propose putative alternative conformations for a target protein with a single known conformation. The method works under the assumption that pairs of structurally similar proteins may also undergo similar conformational changes. Even though the concept itself is not novel, ConTemplate represents the first automated implementation of the idea.

Medina Rodriguez et al [11] developed an algorithm, alleHap, to reconstruct unambiguous haplotypes from parent-offspring pedigree databases with missing family members. Through simulations, they have demonstrated that the algorithm is both fast, achieving optimum performance even with a large number of families, and robust, tolerating inconsistencies of the genotypic data.

To tackle the challenge of detecting low disease risk variants in genome wide association studies (GWAS), Chimusa et al. developed ancGWAS, an algebraic graph based method to identify significant sub-networks underlying ethnic differences in complex disease risk. Using their method, they were able to replicate previous tuberculosis loci, and to introduce novel genes and sub-networks underlying ethnic differences in tuberculosis risk.

In the last oral presentation, Sreedharan et al. [12] presented Oqtans: A Multifunctional

Workbench for RNAseq Data Analysis. To assist the investigation of the abundance of RNA transcripts and their potential differential expression, the workbench enables researchers to set up a computational pipeline for quantitative transcriptome analysis, which can be integrated into the Galaxy framework.

## Award winners

Based on the votes of the SCS delegates, a judging committee awarded four speakers with best oral and poster presentations awards. In the best oral presentation awards the first place went to Han Cheng Lie, for his work “Towards breaking the curse of dimensionality in computational methods for the conformational analysis of molecules” [8]. Second place was for Severine Affeldt, for her work “On the expansion of dangerous gene families in vertebrates” [9].

The first place in the best poster presentation awards was for Maribel Hernandez-Rosales for her work “Simulation of Gene Family histories” [13]. Second place went to Nadezda Kruychkova, for her work “Determinants of protein evolutionary rates in light of ENCODE functional genomics” [14].

## Student Council activities at ISMB

In addition to the ones carried out during the SCS day, the Student Council organizes additional activities aimed at all students and young researchers participating in ISMB/ECCB.

## Career Central and interactive job postings

The Student Council Career Central, reaching in ISMB/ECCB 2013 its third edition, aims to expose students to the experiences and success stories of senior researchers. Dr. Jaap Heringa of Vrije Universiteit Amsterdam gave an overview of the development of his career to the attending young researchers, providing advise based on his experience, and answering questions from the audience.

To facilitate the interaction between job seekers and job advertisers, the Student Council organises an interactive job postings board during ISMB/ECCB. Job offers can be attached to the posting board available at the SC booth in the exhibitors hall, and the advertiser can optionally offer time slots where they would be available for short interviews. Great interest was shown on the interactive system, and as a result many short interviews were carried out.

## Social activities

On the of Student Council's goals is to help its members develop their scientific social network. During ISMB/ECCB, the SC organises two types of social events to achieve this goal. The SC Social Headquarters takes place daily in a location close to the main conference venue. It gives young researchers the opportunity to meet members and leaders of the Student Council and interact in a friendly and relaxed environment. The main social event of the Student Council Symposium took place in a typical “biersalon” in central Berlin, where drinks were offered by the SC to attendees. Both SCS delegates and other young researchers joined the social event, resulting in an attendance of over 70 people.

## Conclusions

This year's number of submissions and participants were roughly equivalent to those of last year's edition in Long Beach. Given the financially turbulent times for science in particular and for the global economy as a whole, we consider this numbers to have been a great success. Without a doubt, the appeal of SCS 2013 is greatly boosted by the outstanding keynote addresses, the high-quality oral presentations, and the broad poster session. Coupled with the rest of the SC-organised activities during ISMB/ECCB, we are happy to report another successful edition of this now long-running event.

Next year's edition, to be held in Boston, United States, will mark the 10^th^ anniversary of the Student Council Symposium. Building on our past and present achievements, we will strive to make the next edition of the Student Council Symposium the best one yet. For further information regarding the Student Council, its events, internships and community, please visit http://www.iscbsc.org.
